# Supplementary material for: An Overview of the Firefly Genus Pygoluciola Wittmer, a Phylogeny of the Luciolinae Using Mitochondrial Genomes, a Description of Six New Species, and an Assessment of a Copulation Clamp in This Genus (Coleoptera: Lampyridae: Luciolinae)
Source: Insects. 2025 Apr 8;16(4):394. doi: 10.3390/insects16040394 (PMC12028155; doi:10.3390/insects16040394)
Supplement: Supplementary file 1 [file insects-16-00394-s001.zip › insects-3514236-supplementary.pdf]

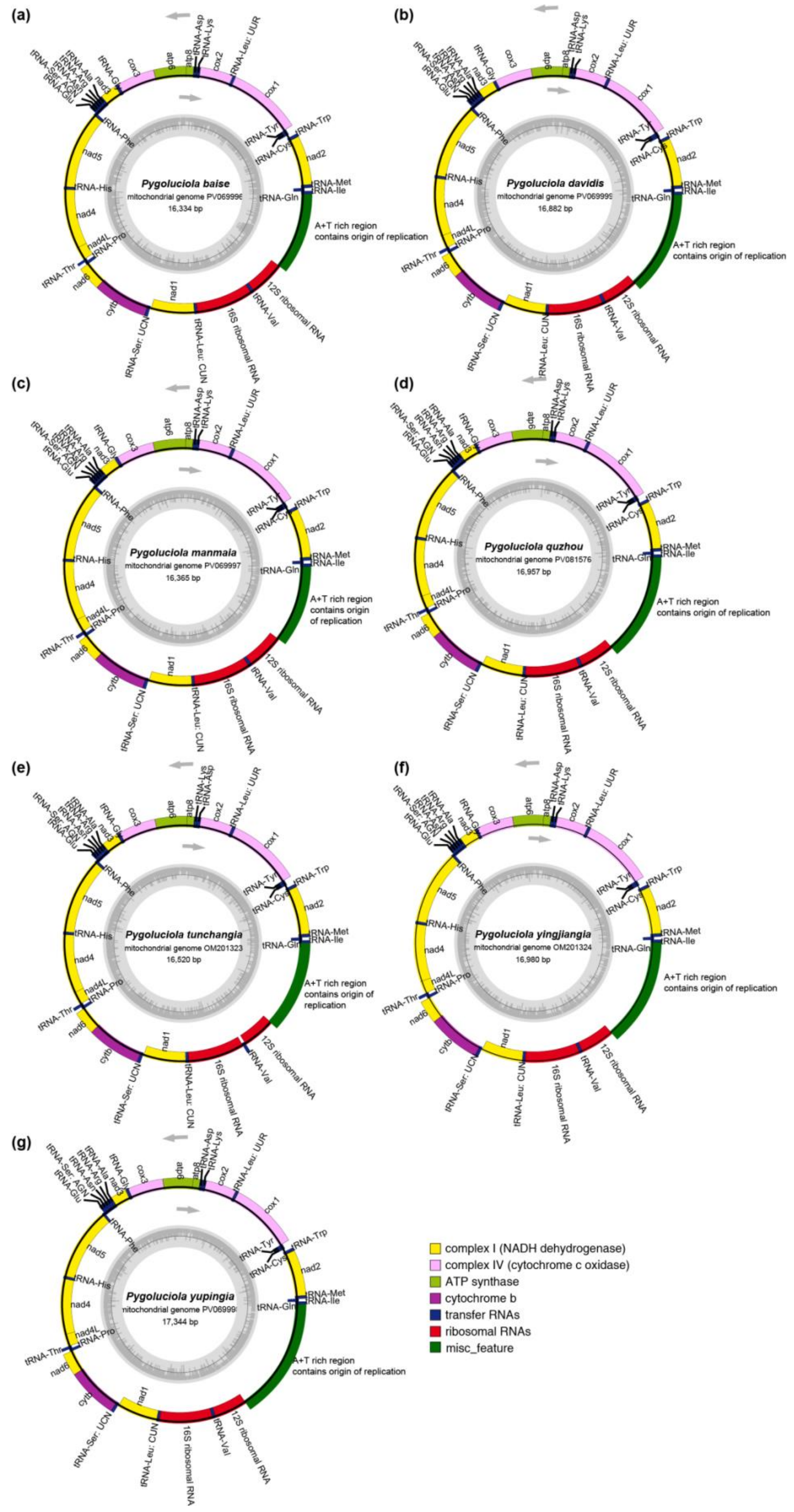

Figure S1. Circular maps of 7 newly sequenced mitogenomes of *Pygoluciola baise* sp. nov., *P. davidis*, *P. manmaia* sp. nov., *P. quzhou* sp. nov., *P. tunchangia* sp. nov., *P. yingjiangia* sp. nov. and *P. yupingia* sp. nov. Genes are represented by different colored blocks, as presented in the legend below the maps. Colored blocks outside of each ring indicate that the genes were on the direct strand, while colored blocks in the rings indicate that the genes were located on the reverse strand.

Table S1**. List of species of *Pygoluciola* Wittmer**

Repeated with modifications and additions from Ballantyne *et al.*[2]

Species	Category	Australia	Malaysia peninsula	Island of Borneo	Myanmar	China	Sri Lanka	Indonesia Java Sumatra	Andaman Islands	India	Bangladesh	Philippines	Location of types	Females associated	Larvae associated
1. *abscondita* (Olivier, 1891)					*								MNHN		
2. *ambita* (Olivier, 1896)								*					MNHN		
3. *bangladeshi* Ballantyne, 2019										*			ANIC	Y	
4. *baise* Fu & Ballantyne sp. nov.						*							HZMAU	Y	
5. *calceata (*Olivier, 1905)							*			*			MNHN		
6. *cowleyi* (Blackburn, 1897)		*											NHML		
7. *davidis* (Olivier, 1895) comb. nov.													MNHN		
8. *dunguna* Nada, 2017 .			*										FRIM	Y	Y
9. *guigliae* (Ballantyne, 1968)				*									BPBM	Y	
10. *hamulata* (Olivier, 1885)				*								*	CMG		
11. *insularis* (Olivier, 1883)									*				MNHN	Y	
12. *kinabalua* (Ballantyne & Lambkin, 2001)				*									NHML	Y	
13. *manmaia* Fu & Ballantyne sp. nov.						*							HZMMAU	Y	
14. *matalangao* Ballantyne, 2019													ANIC	Y	
15. *nitescens* (Olivier, 1905)				*						*			MNHN	Y	
16. *phupan* Ballantyne, 2019													NHML		
17. *qingyu* Fu & Ballantyne, 2008						*							BEIJING	Y	Y
18. *quzhou* Fu & Ballantyne sp. nov.						*							HZMAU	Y	
19. *rammale* Wijekoon et al., 2024							*						COLOMBO	Y	
20. *ruhuna Wijekoon* et al., 2024							*						COLOMBO	Y	
21. *satoi* (Ballantyne, 2008)												*	ZRC	Y	
22. *stylifer* Wittmer, 1939				*									RMNH		
23. *tamarat* Jusoh, 2019													MZUM		
24. *tunchangia* sp. nov.						*							HZMAU	Y	Y
25. *vitalisi* (Pic, 1934)											*		NHML		
26. *wittmeri* (Ballantyne, 1968)				*									BPBM	Y	
27. *yingjiangia* Fu & Ballantyne sp. nov.						*							HZMAU	Y	Y
28. *yupingia* Fu & Ballantyne sp. nov.						*							HZMAU		
